# Anatomical Blueprint of the Sphenoid Sinus in Saudis: A Radiological Observational Perspective

**DOI:** 10.3390/tomography12020026

**Published:** 2026-02-15

**Authors:** Asma F. Al-Muhanna, Musaed A. Al-Fayez, Abdulrahman H. Al-Abdulwahhab, Abdulaziz M. Al-Sharydah, Mohammed Al-Watban, Abdulrazaq Al-Ojan

**Affiliations:** 1 Department of Anatomy, Imam Abdulrahman Bin Faisal University, Dammam 34212, Saudi Arabia; 2 Department of Anatomy, King Saud University, Riyadh 11451, Saudi Arabia; 3 Department of Radiology, Imam Abdulrahman Bin Faisal University, Dammam 34212, Saudi Arabia; 4 Department of Neurosurgery, Imam Abdulrahman Bin Faisal University, Dammam 34212, Saudi Arabia

**Keywords:** sphenoid sinus, paranasal sinuses, septum, computed tomography, volumetric analysis

## Abstract

Background/Objectives: To evaluate and characterize anatomical variations in the sphenoid sinus in the Saudi population using computed tomography (CT). Methods: This retrospective cross-sectional study included patients aged ≥18 years who underwent multi-detector CT (MDCT) of the paranasal sinuses at King Fahd University Hospital between July 2018 and 2023 for different indications. Radiological variables analyzed included sphenoid sinus pneumatization type, presence and number of inter-sphenoid septa, and deviation or attachment to adjacent structures. Results: The data of 2433 patients were analyzed (44.5% males, 55.5% females; mean age 40 ± 15 years). The mean sphenoid sinus volume was 20.4 ± 8.7 cm^3^, significantly larger in males (*p* < 0.001). The most common sinus shape was quadrilateral (33%), whereas the predominant pneumatization pattern was post-sellar (57.1%), followed by sellar (32.1%), pre-sellar (9.2%), and conchal (1.6%). Adjacent-structure pneumatization was frequent, most notably in the greater wing of the sphenoid (47.4%) and pterygoid (39%) processes. Optic-canal protrusion and dehiscence were observed in 13.9% and 4.1%, respectively, whereas carotid canal protrusion occurred in 22.2% and dehiscence in 3.2%. Intra-sinus septation was identified in 97.7% of assessable cases, most commonly as a single septum (59.6%). Several variants showed significant sex-related associations, including sinus volume, anterior clinoid process/posterior clinoid process pneumatization, and dehiscence patterns. Conclusions: CT imaging revealed considerable diversity in the sphenoid-sinus anatomy among the Saudi population. Awareness of these variations, particularly their relationship with critical neurovascular structures, is crucial for radiologists and surgeons to ensure accurate diagnosis and safe surgical planning.

## Simple Summary

We analyzed computed tomography scans of 2433 adult Saudi patients to map variability in sphenoid sinus size, shape, and air-cell extension, and to examine its relationship with nearby critical structures, including the optic nerve and internal carotid artery. Most individuals had a deeply pneumatized “post-sellar” sphenoid sinus extending into adjacent bony processes, with almost all sinuses containing internal septa. Men and women showed differences in the sphenoid sinus and related important structures, which may protrude into the sinus or be partially uncovered. These findings underscore the importance of meticulous, CT-based preoperative assessment.

## 1. Introduction

Although the anatomies of the sphenoid bone, sphenoid sinus, and critical surrounding structures are well described, anatomical variations are frequent and remain clinically significant. Recognizing these differences is crucial for comprehensive preoperative planning, especially in functional endoscopic sinus surgery (FESS) and endoscopic transsphenoidal surgery (ETSS), which are both minimally invasive procedures with low morbidity and mortality [1,2]. Understanding sphenoid sinus morphology and its variants is a cornerstone for surgical safety and efficacy. Multidetector computed tomography (+) allows high-resolution visualization of the paranasal sinuses and is indispensable for neurosurgeons, skull base surgeons, and otorhinolaryngologists [3].

The sphenoid bone is wedge-shaped at the skull base, articulating with various cranial/facial bones and contributing to the cranial fossa, nasal cavity, and orbit. It comprises a central body, greater and lesser wings, and downward-projecting pterygoid processes [1,2,3,4]. The greater wings form part of the middle cranial fossa with the major foramina (rotundum, ovale, and spinosum). In contrast, the lesser wings contain the optic canal and anterior clinoid processes [1,2,3,4], which provide key grooves and canals, with paired sinuses in the sphenoid body divided by a thin septum and opening into the sphenoethmoidal recess. Their important anatomical relationships include superiority to the pituitary gland and optic chiasm, lateral to the cavernous sinus (housing the internal carotid artery and multiple cranial nerves), and being inferior to the nasal cavity [1,2,3,4]. The sphenoid sinus epithelium is thin, pseudostratified, and ciliated columnar, with sparse goblet cells supported by the lamina propria [5], producing limited mucus and showing low infection rates. Its lining is less vascularized, which is attributed to its deep location [6], and the variable thickness of the sinus wall offers structural protection during surgery [7].

Sphenoid sinus pneumatization is classified into conchal, pre-sellar, sellar, and postsellar [8,9,10] pneumatization, which may invade the clivus, clinoid processes, wings, and pterygoid regions, bearing surgical relevance because of its proximity to the optic nerve and internal carotid artery [11,12]. Population studies have shown that the sellar type is prevalent in India, Turkey, Jordan, China, Europe, and America [13,14,15,16,17,18,19], whereas post-sellar dominates in Pakistan, Iran, and Iraq [20,21,22]. The conchal type is rare (typically < 2%) or absent [14,15,16,20]. In 20–50% of cases, pneumatization reaches critical canals (carotid, optic, vidian, and rotundum), increasing the iatrogenic risk [11,12,23]. African and Middle Eastern cohorts showed higher post-sellar rates [23,24,25,26], whereas European and American populations favored the sellar variant [16,17,18]. This wide variability underscores the necessity of meticulous preoperative computed tomography (CT) evaluation to minimize surgical risk [24,27].

The shape, volume, and septal patterns of the sphenoid sinus are highly variable and have direct clinical implications. Rennie et al. classified them by 3D shape (oval, cuboid, triangular, quadrilateral, pentagonal, and hexagonal, with quadrilateral being the most common [3,28], whereas Singh et al. listed spherical, triangular, quadrilateral, pentagonal, and amorphous, again with quadrilateral predominance [28]. Dimensional studies range broadly because of the technique [28,29], and volume estimates by CT/magnetic resonance imaging (MRI) span 3.5–10.3 cm^3^ [30,31,32]. Septation also varies: single, multiple, or absent. It is sometimes attached to vital structures, such as the carotid artery [19,24]. Ethnic comparisons noted smaller, complex Asian sinuses [19], distinct African/Middle Eastern septation and pneumatization [23,24,25], and generally larger, consistent sinuses with fewer septal and access issues in Europeans/Americans [15,16,17,18].

Preoperative imaging, particularly high-resolution imaging, is essential for identifying key variations and optimizing minimally invasive approaches. Doglietto et al. emphasized that outcomes depend on both imaging quality and surgical interpretation [33], with studies showing reduced complications and improved recovery with thorough imaging [34]. Multi-detector CT (MDCT) excels in mapping septa and bony anatomy [18], whereas MRI delineates soft tissue (optic nerve, ICA, and neurovascular elements) [12,15]. Cone beam CT (CBCT) may lower repeat radiation but offers less soft tissue detail [12,34]. Because of frequent canal variability, detailed imaging is critical for anticipating access issues and neurovascular complications [26,35]. FESS/ETSS benefits from recognizing extensive pneumatization (which may ease access or increase risk) versus scant pneumatization (which may restrict corridors), demanding tailored planning [12,15], and ultimately integrating CT/MDCT, CBCT, and MRI with surgical expertise, allowing for individualized management, adaptation to anatomical variants, and safe and effective intervention [12,15,18,26,34].

This retrospective cohort study aimed to identify and describe variations in the sphenoid bone, sphenoid sinus, and related structures, thereby providing insights into the most common anatomical variants observed in the Saudi Arabian population.

## 2. Materials and Methods

### 2.1. Study Design and Setting

This observational study was conducted at King Fahd University Hospital (KFHU) in Al-Khobar, Eastern Province, Saudi Arabia. This study reviewed CT paranasal sinus scans of adult patients to evaluate the anatomical variations in the sphenoid sinus over a 5-year period, from July 2018 to July 2023. Ethical approval was obtained from the Institutional Review Board (IRB). As this was a retrospective imaging-based study with no interventions, the requirement for informed consent was waived, and patient confidentiality was maintained throughout the study.

### 2.2. Participants

**Figure 1:**
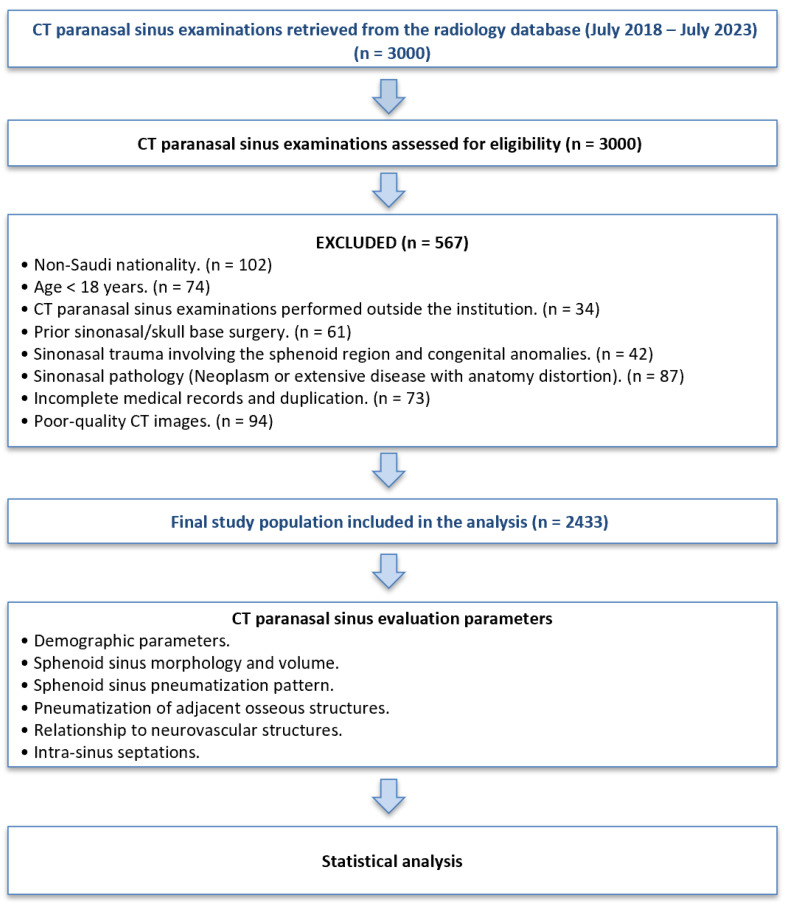
Flowchart illustrating the case selection process, exclusion criteria, and analytical workflow of this retrospective cross-sectional CT study evaluating sphenoid sinus anatomy in the Saudi population.

The study initially identified 3000 patients from the KFHU Radiology Database. After applying the inclusion and exclusion criteria, 2433 patients were included in the final analysis. The study population comprised adult Saudi patients (≥18 years) who had undergone MDCT scans of the paranasal sinuses, with adequate image quality, allowing for detailed evaluation of the sphenoid sinus. Patients were required to have been treated or followed up at KFHU during the study period. The exclusion criteria included patients with congenital craniofacial anomalies, prior sinonasal surgery, sinonasal trauma, benign and malignant sinonasal neoplasms, extensive pathology that distorted the normal sphenoid sinus anatomy, incomplete medical records, or poor-quality CT scans. Patients who underwent imaging outside KFHU or outside the designated study period were excluded (Figure 1).

### 2.3. Variables

The primary dependent variables comprised the anatomical variations in the sphenoid sinus, including the type of pneumatization pattern, sinus shape, volumetric measurements, intra-sinus septation characteristics (presence, number, and position), and their relationship with adjacent neurovascular structures and osseous structures, which included the optic canal, internal carotid artery, vidian canal, foramen rotundum, anterior and posterior clinoid processes, greater wing of the sphenoid, pterygoid processes, and the presence of a spheno-ethmoidal air cell.

Independent variables included demographic data (age and sex). In cases with absent or negligible sphenoid sinus aeration, reliable assessment of certain parameters (such as volume or shape) was not technically feasible; such cases were considered non-assessable only for those specific variables and were excluded from the corresponding analyses.

### 2.4. Data Sources and Measurements

The data were retrieved from the KFHU Radiology Database. Information was collected using a structured data collection sheet designed to capture sociodemographic details, relevant clinical histories, and radiological findings. CT images of the paranasal sinuses were reviewed, and data on sphenoid sinus pneumatization patterns, septation, and extensions into adjacent structures were extracted from the radiologists’ interpretations and verified using the General Electric Picture Archiving and Communication System (PACS).

### 2.5. Imaging Technique

All participants underwent high-resolution CT of the paranasal sinuses using a standardized imaging protocol optimized for the anatomical evaluation of the sphenoid sinus. The imaging studies were independently reviewed by two qualified neuroradiologists (AA, MA), who assessed pneumatization patterns, septation characteristics, and the relationship between the sphenoid sinus and adjacent neurovascular and osseous structures, such as the optic canal and internal carotid artery and presence of spheno-ethimoidal cell. Random sampling was performed to check the consistency of the findings, with subsequent agreement on all results. The Cronbach’s alpha coefficient was 0.949, indicating adequate reliability. The kappa test was conducted to evaluate the significance of the agreement (>80%).

All participants underwent non-contrast high-resolution MDCT of the paranasal sinuses using a SOMATOM Definition Flash scanner (Siemens Healthineers, Erlangen, Germany), a 128-slice dual-source CT system, at King Fahd University Hospital. Image acquisition was performed using a standardized institutional protocol.

Scanning parameters included tube voltage of 120 kVp with automatic tube current modulation (CARE Dose4D). The system provides a gantry rotation time of 0.28 s and a temporal resolution of 75 ms, enabling high-quality imaging with reduced motion artifacts. Data were acquired using submillimetric detector collimation, achieving an isotropic spatial resolution of approximately 0.33 mm, with a table speed of up to 458 mm/s. Images were reconstructed using a high-resolution bone reconstruction algorithm, incorporating Advanced Modeled Iterative Reconstruction for noise reduction and dose optimization. Additional dose-reduction features, including Adaptive Dose Shield, were routinely applied.

Axial images were reconstructed at a slice thickness of 1.0 mm with no interslice gap. Multiplanar reconstructions were generated in the coronal and sagittal planes, oriented perpendicular and parallel to the hard palate. All images were reviewed on a dedicated Siemens PACS workstation using standardized bone window settings.

**Figure 2:**
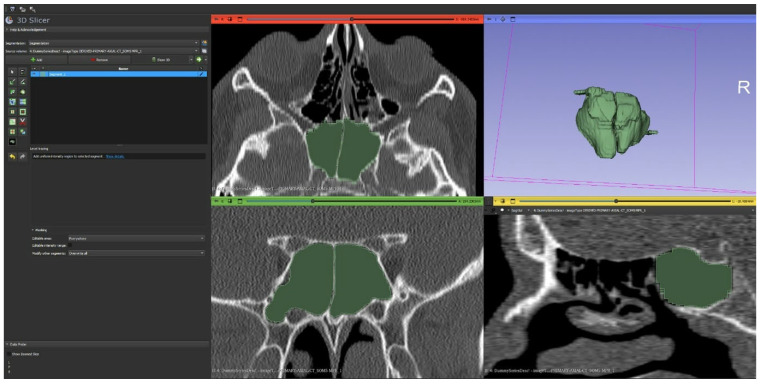
Representative example of sphenoid sinus segmentation, volume measurement, and shape estimation using 3D Slicer software.

For volumetric and morphometric analysis, anonymized DICOM datasets were exported to 3D Slicer software 5.10.0 (United States), a platform originally developed at the Surgical Planning Laboratory, Brigham and Women’s Hospital, and the Massachusetts Institute of Technology (https://www.slicer.org “(accessed on 1 February 2026)”), enabling three-dimensional segmentation and quantitative assessment of sphenoid sinus volume (Figure 2).

### 2.6. Data Collection

Data were systematically extracted from radiology and electronic hospital records. Collected variables included sociodemographic characteristics and detailed radiological parameters, such as the type of sphenoid sinus pneumatization, presence and number of inter-sphenoid septa, and septal deviation or attachment to adjacent structures. Additional variables recorded included sphenoid sinus dimensions, extension into neighboring osseous or neurovascular structures, presence of bony protrusion or dehiscence, and spheno-ethmoidal air cell pneumatization. All CT examinations were independently reviewed and classified by two experienced neuroradiologists to ensure accuracy and consistency of interpretation. Sphenoid sinus volume and shape analyses were performed using 3D Slicer software (Figure 2).

### 2.7. Statistical Analysis

Continuous variables, such as age, were expressed as mean ± standard deviation (SD), along with minimum and maximum values. Categorical variables, including the types of sphenoid sinus pneumatization, septation patterns, and extension into adjacent structures, were reported as frequencies and percentages. Comparisons between groups (e.g., by sex or nationality) were performed using Fisher’s Exact Test for categorical variables and the Mann–Whitney U test for continuous variables. Statistical significance was set at *p* < 0.05. No imputation method was applied to the missing data. Patients with incomplete or poor-quality CT scans were excluded from the final analysis.

## 3. Results

### 3.1. Study Population

A total of 2433 participants were included, representing a broad adult age range with a modest female predominance (Table 1).

### 3.2. Sphenoid Sinus Volume

Sphenoid sinus volumetric analysis was successfully performed in 2407 of the 2433 cases included in the study. Volumetric measurements could not be obtained in 26 cases owing to anatomical limitations. However, the sphenoid sinus volume demonstrates considerable inter-individual variability. A clear sex-related pattern was observed, with males exhibiting significantly larger sinus volumes than females (21.14 vs. 17.81 cm^3^, *p* < 0.001). This difference persisted even after accounting for variations in the age distribution (Table 2).

### 3.3. Sphenoid Sinus Shape

**Figure 3:**
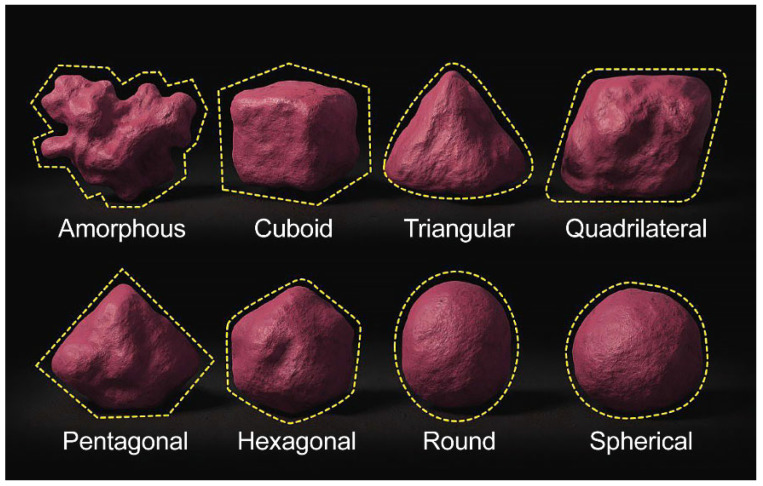
Three-dimensional reconstructions of the sphenoid sinuses illustrating the most common anatomical shapes.

The sphenoid sinuses showed substantial morphological heterogeneity. Although several geometric configurations were identified, the quadrilateral and amorphous shapes were predominant. The morphological distribution differed significantly between sexes; males tended to exhibit more complex or irregular configurations, whereas females more frequently demonstrated the quadrilateral type (Table 3; Figure 3). The sphenoid sinus shape could not be determined in 26 cases owing to insufficient sinus aeration and was therefore reported as non-classifiable.

### 3.4. Pneumatization Patterns

**Figure 4:**
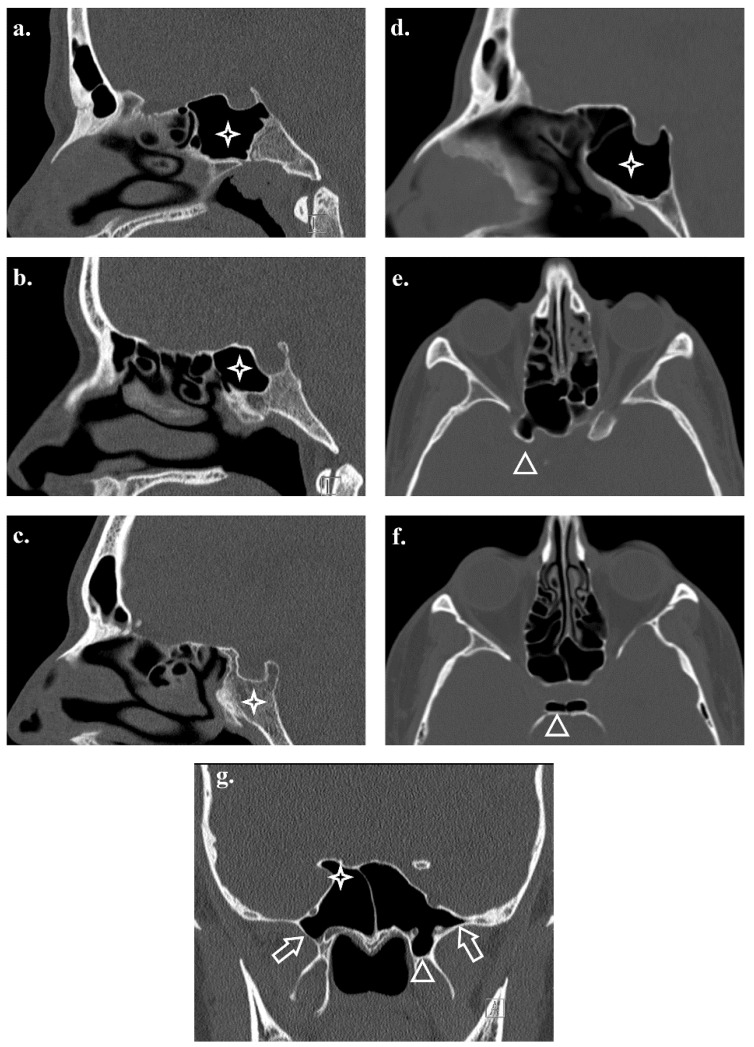
Axial, coronal, and sagittal CT images of the sphenoid sinus illustrating different patterns of pneumatization, Sagittal reconstructions demonstrate: (**a**) sellar type, (**b**) pre-sellar type, (**c**) conchal type, and (**d**) post-sellar type pneumatization (asterisks). Axial CT images show (**e**) pneumatization of the anterior clinoid process and (**f**) bilateral pneumatization of the posterior clinoid processes (arrow heads; anterior angles). The coronal CT image (**g**) demonstrates pneumatization of the bilateral greater wings of the sphenoid (arrows) and the left pterygoid process (arrowhead; anterior angle) and pneumatization of the right anterior clinoid process (asterisk).

Post-sellar pneumatization was the dominant anatomical pattern. Although the sellar and pre-sellar types have also been encountered, the conchal variant remains rare. The pneumatization patterns showed sex-related differences, with males demonstrating a higher propensity for deeper or more extensive pneumatization (Table 3; Figure 4).

### 3.5. Pneumatization of Adjacent Structures

The extension of pneumatization into adjacent osseous structures, including the anterior and posterior clinoid processes, greater wing, and pterygoid processes, varied. These variations exhibited structure- and sex-specific trends; males more commonly demonstrated clinoid and greater-wing involvement, whereas females exhibited higher rates of pterygoid process pneumatization and presence of spheno-ethmoidal air cell. (Table 4; Figure 4).

### 3.6. Relationship to Neurovascular Structures

**Figure 5:**
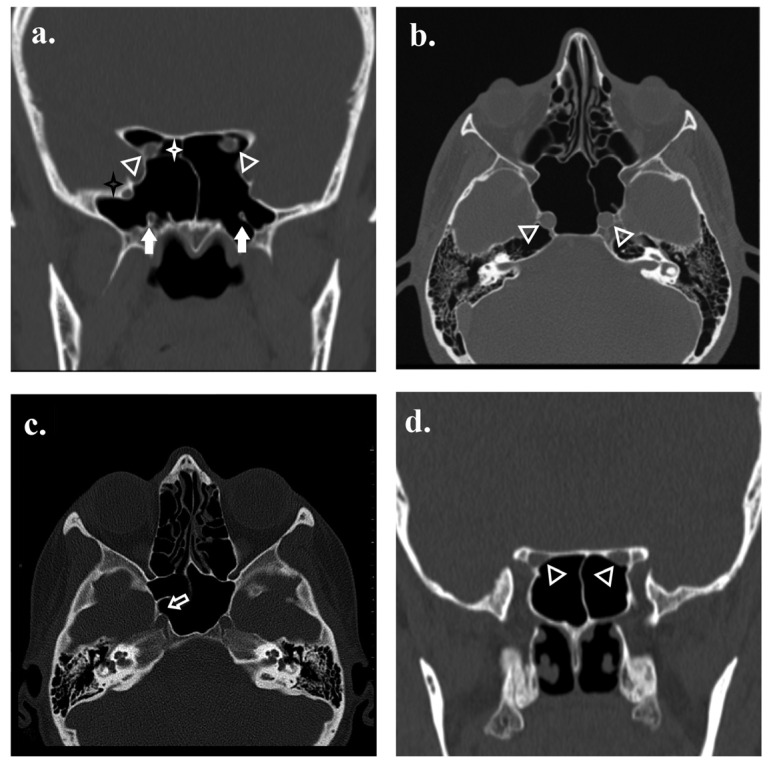
Protrusion and dehiscence of sphenoid sinus–related structures. (**a**) Coronal CT image demonstrates protrusion of the bilateral optic nerves (arrow heads; anterior angles), right foramen rotundum (black asterisk), and bilateral vidian canals (thick white arrows) into the sphenoid sinus, with the presence of a right spheno-ethmoidal air cell (white asterisk). (**b**) Axial CT image showing protrusion of the bilateral carotid canals (arrowheads; anterior angles). (**c**) Axial CT image demonstrating dehiscence of the right carotid canal (arrow). (**d**) Coronal CT image demonstrating dehiscence of both optic canals (arrowheads; anterior angles).

The sphenoid sinus and critical neurovascular structures exhibit considerable anatomical diversity. Variations in the optic canal, carotid canal, vidian canal, and foramen rotundum have been observed at different frequencies. Sex-related differences were structure-dependent; optic canal dehiscence occurred more often in females, whereas vidian canal dehiscence showed a male predominance. Carotid canal variations were relatively infrequent and did not demonstrate meaningful sex-based disparities (Table 4; Figure 5).

### 3.7. Intra-Sinus Septation

**Figure 6:**
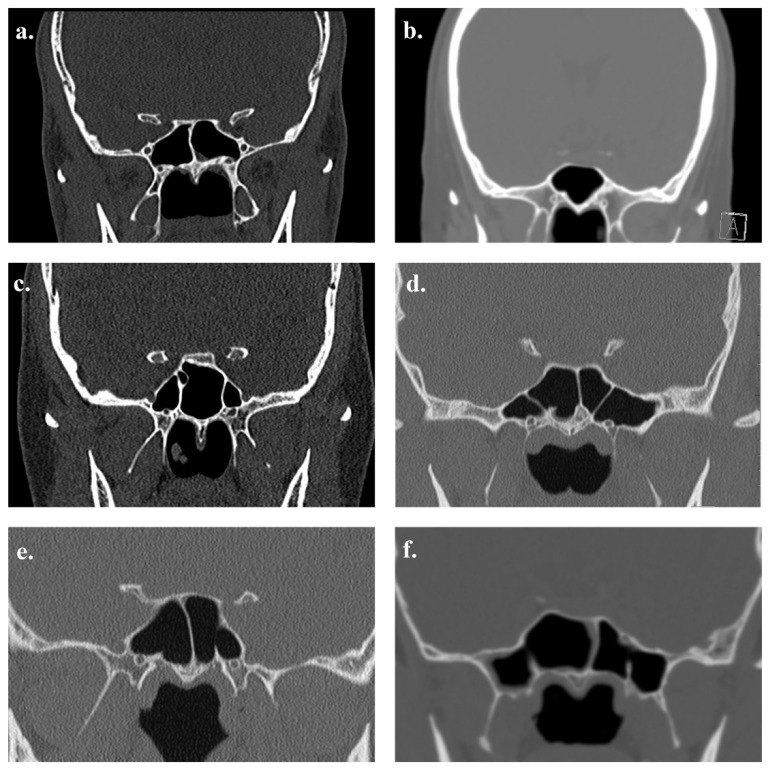
Coronal CT images demonstrating variations in sphenoid inter-sinus septation: (**a**) single central complete septum; (**b**) absent septum; (**c**) two complete septa; (**d**) three complete septa; (**e**) two septa, consisting of a central complete septum and a left-sided incomplete septum; and (**f**) three septa, consisting of a central complete septum with right- and left-sided incomplete septa.

Intra-sinus septation is almost universal. Although most subjects exhibited a single complete septum, multiple septal configurations were observed. Septal number and position varied meaningfully between the sexes; males were more likely to present with a single midline or paramedian septum, whereas females more commonly demonstrated multiple or laterally deviated septa (Table 5; Figure 6).

## 4. Discussion

This study provided a comprehensive analysis of the anatomical variations in the sphenoid sinus in a large Saudi cohort using CT imaging. To our knowledge, this is one of the largest population-based studies in the region to assess sphenoid sinus morphology, morphometry, septal patterns, pneumatization patterns, and their relationships with adjacent neurovascular structures.

Most patients demonstrated a post-sellar type of pneumatization (57.1%), which is consistent with findings from studies in Iran, Pakistan, and Iraq, where the post-sellar and sellar types are predominant [20,21,22]. In contrast, European and American studies have reported the sellar type to be the most common [16,17,18]. The conchal type was rare (1.6%), which is consistent with earlier reports confirming its rarity [14,20,36]. The predominance of the quadrilateral sinus shape (33%) and the frequent occurrence of greater wing (47.4%) and pterygoid process (39%) pneumatization further highlight the variability and surgical relevance of these structures, findings that align with those of earlier morphometric studies [3,12].

The observed optic canal protrusion (13.9%) and dehiscence (4.1%) were slightly lower than those reported in some Asian and European cohorts, which documented optic canal protrusion in 20–30% of cases [12,23]. Carotid canal protrusion (22.2%) and dehiscence (3.2%) were comparable with those reported in previous studies [23,37]. Importantly, vidian canal protrusion (55.9%) and foramen rotundum protrusion (27.9%) were relatively frequent, underscoring the need for meticulous surgical planning, as emphasized in prior anatomical analyses [11,26,27,35].

The spheno-ethmoidal air cell was seen in 33% of cases, a prevalence that is broadly comparable to rates reported in European CT-based studies, while being higher than those described in Asian populations [11,18,26]. This anatomical variant is clinically significant because it frequently lies in close proximity to critical neurovascular structures. As a result, unrecognized spheno-ethmoidal air cells can increase the risk of optic nerve injury or vascular complications during procedures. Careful preoperative CT evaluation is therefore essential to accurately identify this variant and to guide safe surgical planning [26,35].

Sex-related differences in the sphenoid sinus volume and pneumatization patterns were also notable. Males demonstrated significantly larger volumes and higher rates of anterior and posterior clinoid pneumatisation, whereas females showed higher rates of multiple or incomplete septa. These findings are consistent with those of previous volumetric and morphological analyses that have noted sex-linked differences in sinus anatomy [28,35].

Detailed knowledge of sphenoid sinus variations is critical for surgeons performing FESS and ETSS. Variants, such as extensive pneumatization of the clinoid processes or greater wings, increase the likelihood of exposing or injuring critical structures, including the optic nerve and internal carotid artery [6,12]. Similarly, the presence of intra-sinus septa attaching to neurovascular structures may complicate surgical access and increase the risk of iatrogenic injuries [15,37]. In the present study, intra-sinus septation was identified in the vast majority of assessable cases, most commonly as a single septum (59.6%), underscoring the importance of preoperative CT imaging for accurate septal mapping prior to surgery, as previously recommended [12,15].

The strengths of this study include the large sample size and systematic evaluation of a wide range of anatomical parameters using high-resolution CT. This study also provides population-specific data for Saudi Arabia that can serve as a reference for regional surgical practices. This study was limited by its retrospective design and lack of surgical correlation, preventing confirmation of the clinical relevance of the identified anatomical variations. A selection bias may also be present because the sample included only patients who underwent CT for clinical indications, which may not represent the general population. In addition, variations in imaging protocols and scanner parameters may have influenced the detection of subtle bony changes. Future prospective studies incorporating standardized imaging, intraoperative correlation, and the inclusion of asymptomatic cohorts are recommended to validate these findings and better define their surgical and clinical implications.

## 5. Conclusions

This study confirmed that the sphenoid sinus anatomy exhibits substantial variability in the Saudi population, with post-sellar pneumatization being the most common pattern. The frequent occurrence of sinus extension into adjacent structures and the high prevalence of septa emphasize the critical importance of preoperative CT evaluation to minimize complications in FESS and ETSS. These findings contribute to the global body of literature while providing region-specific data for safer surgical planning.

## Figures and Tables

**Table 1:** Descriptive statistics of the study participants.

Central Tendencies	Items	
	Age (*n* = 2433)	Sphenoid Sinus Volume (*n* = 2407) *
Mean ± SD	40.04 ± 15	20.39 ± 8.7
Median (IQR)	38 (28, 52)	19.23 (14.8, 24.9)
Mode	23	17.96
Min.–Max. (Range)	18–89 (72)	3.06–57.65 (54.6)
**Items**	***n* (%)** **2433 (100)**	
**Sex**		
Male	1083 (44.5)	
Female	1350 (55.5)	
**Sphenoid sinus shape**		
Amorphous	696 (28.6)	
Cuboid	303 (12.5)	
Triangular	221 (9.1)	
Quadrilateral	802 (33)	
Pentagon	201 (8.3)	
Hexagon	58 (2.4)	
Round	12 (0.5)	
Spherical	114 (4.7)	
Not classifiable **	26 (1.1)	
**Sphenoid sinus pneumatization**		
Conchal	40 (1.6)	
Pre-sellar	224 (9.2)	
Sellar	780 (32.1)	
Post-sellar	1389 (57.1)	
**ACP pneumatization**		
Yes	627 (25.8)	
No	1806 (74.2)	
**PCP**		
Yes	70 (2.9)	
No	2363 (97.1)	
**GWS**		
Yes	1153 (47.4)	
No	1280 (52.6)	
**PP**		
Yes	948 (39)	
No	1485 (61)	
**Optic canal protrusion**		
Present	337 (13.9)	
Absent	2096 (86.1)	
**Optic canal dehiscence**		
Present	100 (4.1)	
Absent	2333 (95.9)	
**Carotid canal protrusion**		
Present	539 (22.2)	
Absent	1894 (77.8)	
**Carotid canal dehiscence**		
Present	78 (3.2)	
Absent	2355 (96.8)	
**Vidian canal protrusion**		
Present	1361 (55.9)	
Absent	1072 (44.1)	
**Vidian canal dehiscence**		
Present	876 (36)	
Absent	1557 (64)	
**Foramen rotundum protrusion**		
Present	679 (27.9)	
Absent	1754 (72.1)	
**Foramen rotundum dehiscence**		
Present	138 (5.7)	
Absent	2295 (94.3)	
**Spheno-ethmoidal air cell**		
Present	803 (33)	
Absent	1630 (67)	
**Intra-sinus septa**		
Absent	28 (1.2)	
Present—Complete	2218 (91.2)	
Present—Incomplete	73 (3)	
Present—Complete and incomplete	88 (3.6)	
Non-assessable ***	26 (1.1)	
Intra-sinus septum number (*n* = 2379) ****		
One	1417 (59.6)	
Two	470 (19.8)	
Three	396 (16.6)	
Four	82 (3.4)	
Five	14 (0.6)	
Intra-sinus one septa position (*n* = 1417) *****		
Central	442 (31.2)	
Right side	480 (33.9)	
Left side	495 (34.9)	

ACP, anterior clinoid process; PCP, posterior clinoid process; GWS, greater wing of sphenoid; PP, pterygoid process. * Sphenoid sinus volume measurements were available in 2407 cases. Volumetric analysis was not feasible in 26 cases owing to absent or negligible sinus air space. ** Sphenoid sinus shape could not be reliably classified owing to absent or negligible sinus air space. *** Intra-sinus septation was assessed only in cases with adequate sinus aeration; cases with absent or negligible air space were classified as non-assessable. **** Septum number was assessed only in cases with classifiable intra-sinus septation; cases without septa (*n* = 28) and non-classifiable cases (*n* = 26) were excluded. ***** Septum position analysis was restricted to cases with a single dominant septum; cases with multiple septa or non-classifiable septation were excluded.

**Table 2:** Association between sex and quantitative variables (age and sphenoid sinus volume).

Items *n* = 2433	Male Median (IQR)	Female Median (IQR)	*p* Value ^a^
**Age (Years)**	36 (28, 52)	39 (29, 52)	0.175
**Sphenoid sinus volume (cm^3^) †**	21.14 (17.2, 26.25)	17.81 (12.9, 23.8)	<0.001 *

^a^ *p*-value for the Mann–Whitney test. * Statistically significant < 0.05. † Sphenoid sinus volume analysis was performed in 2407 cases with a measurable sinus air cavity.

**Table 3:** Association between sex and sphenoid sinus shape and pneumatization.

Items	Male (*n* = 1083) *N* (%)	Female (*n* = 1350) *N* (%)	*p*-Value
**Sphenoid sinus shape**			
Amorphous	324 (29.9)	372 (27.6)	<0.001 ^a,^*
Cuboid	138 (12.7)	165 (12.2)	
Triangular	92 (8.5)	129 (9.6)	
Quadrilateral	328 (30.3)	474 (35.1)	
Pentagon	73 (6.7)	128 (9.5)	
Hexagon	28 (2.6)	30 (2.2)	
Round	0 (0)	12 (0.9)	
Spherical	74 (6.8)	40 (3)	
Not classifiable	12 (1.1)	14 (1)	
Total (*n* = 2407)	1083 (100)	1350 (100)	
**Sphenoid sinus pneumatization**			
Conchal	33 (3.0)	7 (0.5)	<0.001 ^b,^*
Pre-sellar	85 (7.9)	139 (10.3)	
Sellar	329 (30.4)	451 (33.4)	
Post-sellar	636 (58.7)	753 (55.8)	
Total (*n* = 2433)	1083 (100)	1350 (100)	
**ACP pneumatization**			
Yes	325 (30)	302 (22.4)	<0.001 ^b,^*
No	758 (70)	1048 (77.6)	
Total (*n* = 2433)	1083 (100)	1350 (100)	
**PCP**			
Yes	58 (5.4)	12 (0.9)	<0.001 ^b,^*
No	1025 (94.6)	1338 (99.1)	
Total (*n* = 2433)	1083 (100)	1350 (100)	
**GWS**			
Yes	538 (49.7)	615 (45.6)	0.045 ^b,^*
No	545 (50.3)	735 (54.4)	
Total (*n* = 2433)	1083 (100)	1350 (100)	
**PP**			
Yes	377 (34.8)	571 (42.3)	<0.001 ^b,^*
No	706 (65.2)	779 (57.7)	
Total (*n* = 2433)	1083 (100)	1350 (100)	
**Spheno-ethmoidal air cell**			
Yes	260 (24.0)	543 (40.2)	<0.001 ^b,^*
No	823 (76.0)	807 (59.8)	
Total (*n* = 2433)	1083 (100)	1350 (100)	

^a^ *p*-value for the Monte Carlo test. ^b^ p-value for the chi-square test. * Statistically significant at *p* < 0.05. ACP, anterior clinoid process; PCP, posterior clinoid process; GWS, greater wing of sphenoid; PP, pterygoid process. “Not classifiable” refers to cases with absent or negligible sphenoid sinus air space, precluding reliable shape classification.

**Table 4:** Association between sex and sphenoid sinus pneumatization involvement of adjacent structures.

Items *n* = 2433	Male *N* (%)	Female *N* (%)	*p*-Value ^1^
**Optic canal protrusion**			
Present	157 (14.5)	180 (13.3)	0.409
Absent	926 (85.5)	1170 (86.7)	
**Optic canal dehiscence**			
Present	30 (2.8)	70 (5.2)	0.003 *
Absent	1053 (97.2)	1280 (94.8)	
**Carotid canal protrusion**			
Present	257 (23.7)	282 (20.9)	0.093
Absent	826 (76.3)	1068 (79.1)	
**Carotid canal dehiscence**			
Present	31 (2.9)	47 (3.5)	0.389
Absent	1052 (97.1)	1303 (96.5)	
**Vidian canal protrusion**			
Present	595 (54.9)	766 (56.7)	0.374
Absent	488 (45.1)	584 (43.3)	
**Vidian canal dehiscence**			
Present	442 (40.8)	434 (32.1)	<0.001 *
Absent	641 (59.2)	916 (67.9)	
**Foramen rotundum protrusion**			
Present	291 (26.9)	388 (28.7)	0.307
Absent	792 (73.1)	962 (71.3)	
**Foramen rotundum dehiscence**			
Present	43 (4)	95 (7)	0.001 *
Absent	1040 (96)	1255 (93)	

^1^: *p*-value for Chi-square test. * Statistically significant < 0.05.

**Table 5:** Association between sex and Intra-sinus septum presence, completeness, number, and position.

Items	Male *N* (%)	Female *N* (%)	*p*-Value
**Intra-sinus septa**			
Absent	19 (1.8)	9 (0.7)	<0.001 ^1,^*
Present—Complete	1024 (94.6)	1194 (88.4)	
Present—Incomplete	14 (1.3)	59 (4.4)	
Present—Complete and incomplete	14 (1.3)	74 (5.5)	
Non-assessable *	12 (1.1)	14 (1)	
Total (*n* = 2433)	1083 (100)	1350 (100)	
**Intra-sinus septum number**			
One	618 (58.8)	799 (60.2)	<0.001 ^2,^*
Two	215 (20.4)	255 (19.2)	
Three	186 (17.7)	210 (15.8)	
Four	28 (2.7)	54 (4.1)	
Five	5 (0.5)	9 (0.7)	
Total (*n* = 2379) **	1052 (100)	1327 (100)	
**Intra-sinus septum position**			
Central	221 (35.8)	221 (27.7)	0.025 ^1,^*
Right side	165 (26.7)	315 (39.4)	
Left side	232 (37.5)	263 (32.9)	
Total (*n* = 1417) ***	618 (100)	799 (100)	

^1^: *p*-value for chi-square test; ^2^: *p*-value for Monte Carlo test. * Statistically significant at *p* < 0.05. ** Septum number was assessed only in cases with classifiable intra-sinus septation; cases without septa (*n* = 28) and non-assessable septation (*n* = 26) were excluded. *** Denominator (*n* = 1417) correctly reflects single-septum cases only.

## Data Availability

The datasets generated and/or analysed during the current study are not publicly available due to confidentiality of subjects but are available from the corresponding author on reasonable request.

## Abbreviations

The following abbreviations are used in this manuscript:

CT	Computed Tomography
MDCT	Multi-Detector Computed Tomography
FESS	Functional Endoscopic Sinus Surgery
ETSS	Endoscopic Transsphenoidal Surgery
MRI	Magnetic Resonance Imaging
CBCT	Cone Beam Computed Tomography
KFHU	King Fahd University Hospital
SD	Standard Deviation
